# Toosendanin, a late-stage autophagy inhibitor, sensitizes triple-negative breast cancer to irinotecan chemotherapy

**DOI:** 10.1186/s13020-022-00605-8

**Published:** 2022-05-06

**Authors:** Shuang Zhang, Yu Dong, Xiuping Chen, Chris Soon Heng TAN, Min Li, Kai Miao, Jia-Hong Lu

**Affiliations:** 1 State Key Laboratory of Quality Research in Chinese Medicine, Institute of Chinese Medical Sciences, University of Macau, Taipa, 999078 Macau SAR China; 2 Guangdong-Hong Kong-Macau Joint Lab on Chinese Medicine and Immune Disease Research, University of Macau, Taipa, 999078 Macau SAR China; 3 Department of Chemistry, College of Science, Southern University of Science and Technology, Shenzhen, 518055 China; 4 Mr. & Mrs. Ko Chi-Ming Centre for Parkinson’s Disease Research, School of Chinese Medicine, Hong Kong Baptist University, Hong Kong SAR, China; 5 MOE Frontier Science Centre for Precision Oncology, University of Macau, Taipa, Macau SAR China

**Keywords:** Autophagy inhibition, Toosendanin, Triple-negative breast cancer, Lysosome, Irinotecan, SN-38, Apoptosis

## Abstract

**Background:**

Triple-negative breast cancer (TNBC) is a highly aggressive subtype of breast cancer that develops resistance to chemotherapy frequently. Autophagy has been reported as a pro-survival response to chemotherapeutic drugs in TNBC, and suppression of autophagy can be a strategy to overcome drug resistance.

**Methods:**

The efficacy of toosendanin (TSN) in blocking autophagy flux was measured by western blot analysis of autophagy markers, and the fluorescent imaging of RFP-GFP-LC3 probe. The co-localization of autophagosomes and lysosomes was analyzed by fluorescent imaging. Then, lysosome function was determined by measuring the lysosomal pH value and the activity of lysosomal hydrolytic proteases. For in vitro study, human triple-negative breast cancer MDA-MB-231 and MDA-MB-436 cell lines were used for evaluating the anti-proliferative effect. For in vivo study, the RFP-GFP-LC3 MDA-MB-231 xenograft nude mice received intraperitoneal injection of irinotecan (10 mg/kg), TSN (0.5 mg/kg) or a combination, and the autophagy activity and cell apoptosis were determined in tumor tissue. The degree of pathological injury of tissue was evaluated by liver index.

**Results:**

The natural autophagy inhibitor TSN, a triterpenoid extracted from *Melia toosenda* Sieb. *et* Zucc, potently inhibited late-stage autophagy in TNBC cells. This effect was achieved via elevating lysosome pH rather than blocking the fusion of autophagosomes and lysosomes. We further investigated the effects of TSN on the in vitro and in vivo TNBC models, in combination with chemotherapeutic drug irinotecan (or its active metabolite 7-ethyl-10-hydroxycamptothecin), a topoisomerase I inhibitor showing therapeutic potential for TNBC. The data showed that TSN blocked 7-ethyl-10-hydroxycamptothecin (SN-38)/irinotecan-induced protective autophagy, and significantly induced apoptosis in TNBC cells and tumor xenograft models when compared to SN-38/irinotecan alone group.

**Graphical Abstract:**

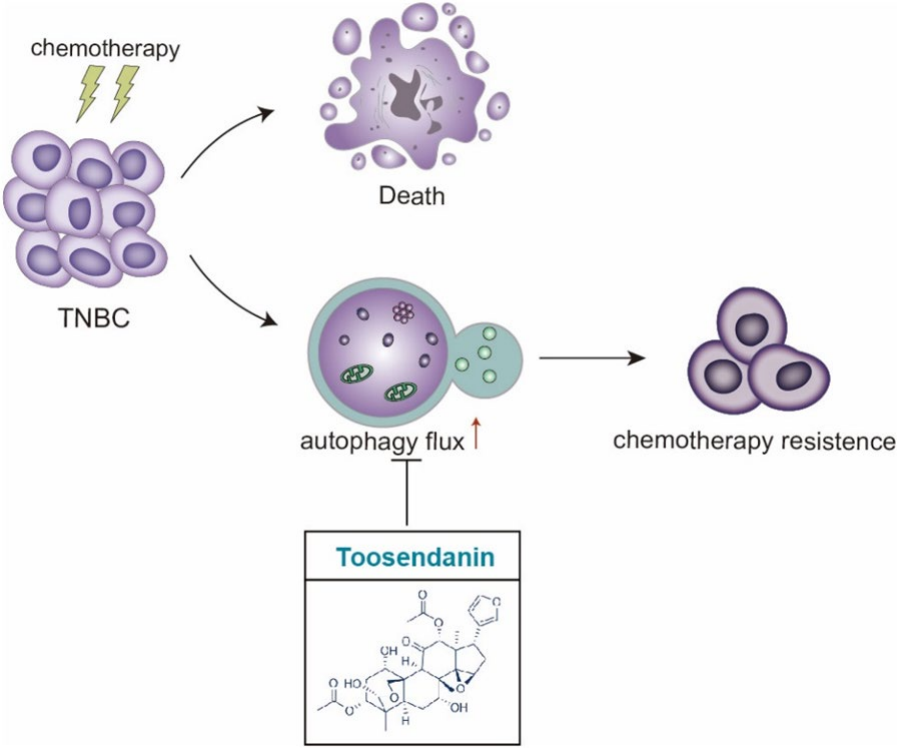

**Supplementary Information:**

The online version contains supplementary material available at 10.1186/s13020-022-00605-8.

## Introduction

TNBC is an aggressive subtype that constitutes 12–18% of breast cancer clinical cases [1, 2]. Because TNBC cells lack the estrogen receptor (ER), progesterone receptor (PR), and human epidermal growth factor receptor 2 (HER2), they are not accessible for hormone or anti-HER2 therapy. Compared to hormone receptor-positive or HER2-positive subtypes, TNBC patients displayed poorer response to anti-cancer treatment, and faster progress of tumor metastasis [2, 3]. To date, chemotherapy remains the standard treatment approach for TNBC [1]. However, 30–50% of TNBC patients rapidly become resistant to chemotherapy, which leads to the failure of treatment [4].

As a cellular survival mechanism to degrade and recycle misfolded proteins and damaged organelles, autophagy is highjacked by cancer cells to overcome metabolic stress and develop drug resistance [5, 6]. In tumor cells with defects in apoptosis, autophagy allows prolonged survival. Accumulation of evidence reveals that inhibition of autophagy can be an effective therapeutic strategy in combination with chemotherapy [7]. At present, only two autophagy inhibitors, chloroquine (CQ) and hydroxychloroquine (HCQ) have been tested in patients, either alone or in combination with chemotherapy. However, due to the high working concentration and lack of specific molecular target(s), CQ may cause side effects, such as visual impairment, gastrointestinal discomfort, headache, and pruritus [8]. Therefore, the development of novel autophagy inhibitor (s) for the cancer treatment has considerable clinical significance.

TSN, a triterpenoid extracted from the root bark of *Melia toosendan* Sieb. *et* Zucc, has been used as an insecticide in China for decades. Recent studies have revealed the anti-tumor effects of TSN on various human cancer cells such as osteosarcoma, lung cancer, and colorectal cancer [9–12] by modulating mitogen-activated protein kinases pathway, epithelial–mesenchymal transition, and estrogen receptor β. Zhang et al. [13] firstly reported that TSN exhibited inhibition function on TNBC growth at the nanomolar level, and the capability may be related to inducing necrosis, apoptosis and autophagy. However, specific mechanisms involved in the anti-tumor effect of TSN on TNBC are still unclear. In the present study, we reported that TSN potently inhibited auto-lysosome maturation, leading to the accumulation of autophagosomes in TNBC cells. This effect was due to inhibiting acidification of lysosome and impairing the lysosomal hydrolytic function rather than blocking the fusion of autophagosomes and lysosomes.

Irinotecan is a topoisomerase I inhibitor which suppresses tumor growth by causing DNA double-strand breaks, and is indicated for the treatment of solid tumors including colorectal and lung cancer [14]. Recent studies highlighted the potential of irinotecan in the TNBC treatment. However, acquired drug resistance currently limits its clinical application. Paillas et al. [15] have proved that SN-38 could induce survival-promoting autophagy depending on mitogen-activated protein kinase 14 (MAPK14).

In this research, we found that TSN sensitized TNBC cells to SN-38/irinotecan-induced cytotoxicity both in vitro and in vivo. Our findings thus demonstrated that the novel late-stage autophagy inhibitor TSN may represent a therapeutic potential for TNBC, in combination with chemotherapy drugs including irinotecan.

## Materials and methods

### Antibodies and reagents

The chemicals: Toosendanin, irinotecan (SHANGHAI XIANDING BIOLOGICAL SCIENCE & TECHNOLOGY CO. LTD, HN057, 136572-09-3), Torin 1 (LC Laboratories, T-7887), SN-38, chloroquine, baflomycin A1 (Sigma-Aldrich, H0165, C6628, 19-148), LysoTracker Red DND-99, LysoSensor Yellow/Blue DND-99 (Thermo Fisher, M22425, L7545).

The antibodies: microtubule-associated protein 1 light chain 3 (LC3, Novus, NB100-2220), Sequestosome 1 (SQSTM1, ABclonal, A11250), Lysosome-associated membrane protein 1 (LAMP1), Cleaved caspase 3, and GAPDH (Cell Signaling Technology, 9091, 9661, D16H11), Cathepsin B, Cathepsin D (Santa Cruz, SC365558, SC-6486). Alexa Fluor 488-, 555- or 647-conjugated goat anti-rabbit and goat anti-mouse antibodies purchased from Invitrogen (A-11034, A-21422 and A-32733).

### Cell culture

MDA-MB-231, MDA-MB-436, and BT-549 cells were obtained from the American Type Culture Collection and cultured in Dulbecco’s modified Eagle’s medium (DMEM) supplemented with 10% fetal bovine serum (Gibco, 10100-147). MDA-MB-231 cells stably expressing RFP-GFP-LC3 maintained in DMEM with 10% FBS and 0.2 μg/μL G418. All the mediums were supplemented with 1% penicillin and streptomycin (Thermo Fisher Scientific, 12100046). Cells were incubated at 37 °C in a humid 5% CO_2_:95% air environment.

### Cell viability assay

The 3-(4,5-dimethylthiazol-2-yl)-2,5-diphenyl-2H-tetrazolium bromide (MTT) staining method as described by Mosmann [16] was used with minor modifications. Cells were seeded in 96-well plates at 5000 per well and were treated for 24 to 48 h depending on experimental conditions. Twenty microlitre MTT (5 μg/μL) was added in each well and incubated for 4 h. 100 μL dimethyl sulfoxide (DMSO) was loaded into each well to dissolve the formazan and then optical density (O.D.) value was measured by microplate reader at 570 nm. The cell viability was calculated as the ratio of (experimental group - blank) vs (control group- blank).

### Transfections

Transfection was achieved using Lipofectamine 3000 Transfection Reagent (Invitrogen, L3000) according to the manufacturer's protocol. Cells were transfected with plasmids encoding red fluorescent protein (RFP)-LC3, green fluorescent protein (GFP)- LC3, RFP-GFP-LC3, and mCherry-LAMP1. After 24 h incubation, the transfection mixture was removed and replaced with fresh complete medium.

### Western blots

Cell proteins were extracted using ice-cold radio-immunoprecipitation assay buffer (RIPA, Cell Signaling Technology, 9806) with complete protease inhibitor mixture (Roche Applied Science, 04693124001). Protein was separated by gel electrophoresis in 10–15% SDS-polyacrylamide gels and subsequently transferred onto polyvinylidene difluoride (PVDF) membranes (Bio-Rad, 1704156). Following blocking with TBST (Tris-buffered saline with 0.1% Tween-20) buffer containing 5% (w:v) nonfat milk powder (Bio-Rad, 1706404), the blots were probed with the corresponding primary antibodies and secondary antibody. Blots were visualized using the Pierce ECL kit (Pierce, 32106) and ChemiDoc MP Imaging System (Bio-Rad, 12003154).

### Immunofluorescence

After treatment, MDA-MB-231, MDA-MB-436 cells were fixed in 4% paraformaldehyde (Shanghai Sangon Biotech, E672002-0500) for 15 min at room temperature (RT) and blocked in 5% Bovine Serum Albumin Standard (BSA) for 1 h. Subsequently, cells were incubated with anti-LAMP1 (1:100) antibodies overnight at 4 °C and then incubated with the appropriate secondary antibodies for 1 h at RT. Tumor tissues were snap-frozen in optimal cutting temperature (OCT) embedding medium (Tissue-Tek) and sectioned. Cryosections of tumors (5 μm thickness) were also fixed in 4% paraformaldehyde for 15 min at RT and blocked in 5% BSA for 1 h. Nuclei were stained with Hoechst 33258 for 5 min. Fluorescence photos were taken using a confocal laser-scanning microscope. Different fields of view (> 5 regions) were analyzed on the confocal laser-scanning microscope for each labeling condition, and representative results were shown.

### Lysosomal pH measurements

Lysosomal pH was measured by LysoSensor Yellow/Blue DND-160 (Invitrogen, L7545) staining according to the manufacturer’s protocol. MDA-MB-231, MDA-MB-436, and BT-549 cells were plated on the 96-well culture plates. After treatment, cells were loaded with 2 µM LysoSensor Yellow/Blue DND-160 for 30 min at 37 °C and washed twice using PBS. After that, 100 μL HBSS (containing Mg^2+^, Ca^2+^) was added to each well for further detection. Fluorescence emitted at 440/540 nm was measured by a microplate reader (Molecular devices FlexStation 3) in response to excitation at 329 nm and 384 nm, respectively.

### Annexin-V/propidium iodide (PI) dual staining assay

A quantitative assessment of apoptosis cells was performed using the Annexin V-FITC Apoptosis Detection Kit (Beyotime Biotechnology, C1062M). In short, the cells were cultured in a 6-well cell culture plate and treated with TSN, SN-38 alone, or in combination. Then cells were collected, washed with cold PBS, and resuspended in binding buffer (1 × 10^6^ cells/mL). After 100 µL of cells was transferred to a tube, added 5 µL of FITC-conjugated Annexin V (Annexin V-FITC) and 2 µL of PI and incubated for 15 min at RT in the dark. The stained cells were analyzed by the flow cytometer (BD LSR Fortessa™ Flow Cytometer). Data of 10,000 cells were collected in each data file. Four different populations of cells were easily distinguished: un-labelled (viable cells), Annexin V-FITC positive (early apoptotic), PI positive (necrotic), and Annexin V-FITC/PI positive (late apoptotic/necrotic cells). The fluorescence distribution was displayed as a two-color dot plot analysis, and the cells in each quadrant were determined.

### Lactate dehydrogenase (LDH) release assay

LDH Cytotoxicity Assay Kit (Beyotime Biotechnology, C0016) was used to evaluate cell death. Specifically, the LDH working solution was prepared by 10 μL lactic acid solution, 10 μL INT solution (1×), 10 μL enzyme solution, with a total of 30 μL per well. The cell culture 96-well plate was centrifuged at 400*g* for 5 min. The supernatant of each well (60 μL) was added into the corresponding well of a new plate. The LDH working fluid was mixed with the supernatant, incubated at RT for 30 min, and then the absorbance was read at 490 nm.

### Measurement of intracellular ROS levels

The intracellular ROS levels were measured using a Reactive Oxygen Species Assay Kit (Beyotime Biotechnology, China). 2ʹ, 7ʹ-dichlorofluorescein-diacetate (DCFH-DA), which is easily oxidized to fluorescent dichlorofluorescein (DCF) by intracellular ROS, and therefore, the intracellular ROS levels were quantified. Briefly, the cells were seeded in 24-well plates and exposed to SN-38 (0.1 μM) and/or TSN (0.1 μM). Following the treatment, the cells were incubated with DCFH-DA for 20 min at 37 °C and then observed using confocal laser-scanning microscope (488 nm excitation and 525 nm emission).

### Xenograft assay

Nude mice aged 4–5 weeks old were obtained from the Faculty of Health Sciences, University of Macau, and fed with a standard animal diet and water. Animal research was approved by the Animal Ethics Committee in University of Macau (UMARE-036-2019). MDA-MB-231 cells stably expressing RFP-GFP-LC3 were suspended in a 1:1 ratio in serum-free DMEM medium with a matrigel basement membrane matrix (Corning, 356231) and inoculated subcutaneously (5 * 10^6^/site) in the right armpit. The tumor diameters were measured and the tumor volume (mm^3^) was calculated with caliper as follows: Volume = (shortest diameter)^2^ × (longest diameter)/2. The volume between 80 and 120 mm^3^ was considered as a successful establishment of the model. Irinotecan (10 mg/kg, every 6 days) and TSN (0.5 mg/kg, every 2 days) were dissolved in intralipid and injected into the mice intraperitoneally alone or together (*n* = 7). Intralipid was used as the vehicle control. Tumor size and body weight are recorded every 2 days or 3 days. Mice were sacrificed 28 days after medication. Organs were collected and tumors were excised, and either formalin-fixed or flash-frozen at − 80 °C until further use.

### Statistical analysis

Each experiment was performed at least 3 times, and the results are presented as mean ± SD. One-way analysis of variance (ANOVA) was followed by Turkey as post hoc tests using the Sigma Plot 11.0 software package. A probability value of p < 0.05 was considered statistically significant.

## Results

### TSN caused LC3-II accumulation and suppressed autophagic degradation

**Fig. 1:**
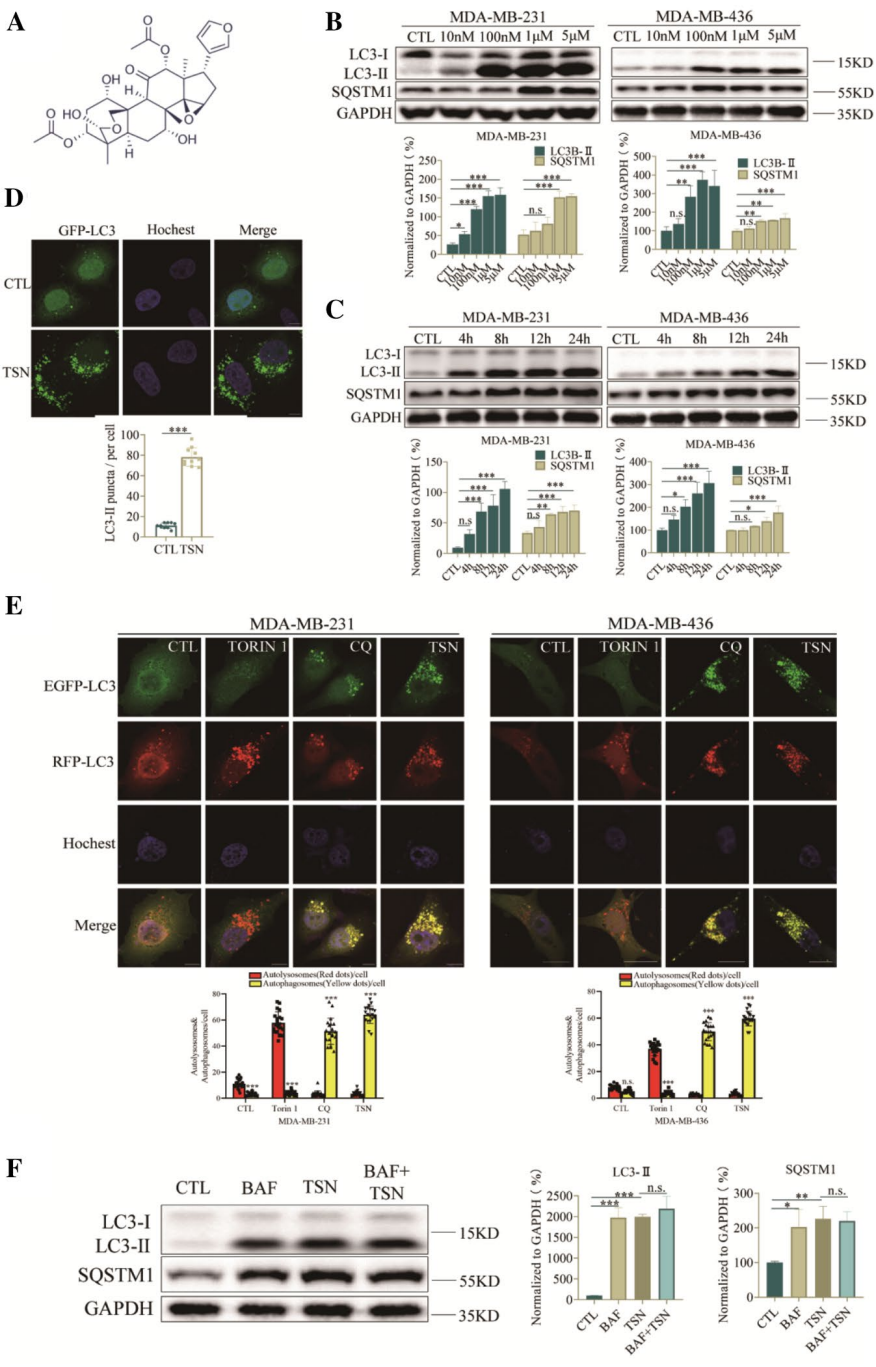
TSN caused LC3-II accumulation stability and suppressed autophagic degradation. **A** The chemical structure of TSN with a molecular weight of 574.62 g/mol (CAS Number: 58812-37-6). **B** Western blot analysis of LC3-II and SQSTM1 levels in MDA-MB-231 and MDA-MB-436 cells treated with the indicated concentrations (0.01–5 μM) of TSN for 24 h. **C** Western blot analysis of LC3-II and SQSTM1 levels in MDA-MB-231 and MDA-MB-436 cells treated with TSN (1 μM) at the time points indicated (0, 4, 8, 12, 24 h). **D** MDA-MB-231 cells were transiently transfected with GFP-LC3 plasmid. The images were captured under Leica TCS SP8 confocal laser scanning microscope after treatment of TSN (1 μM) or DMSO for 24 h. Scale bar: 10 μm. 20 cells in each group were counted for data analysis. **E** Cells were transfected with a tandem fluorescent-tagged LC3 (tfLC3), and were exposed to TSN (1 μM), CQ (30 μM) and Torin1 (100 nM) as indicated. The co-localization of GFP-LC3 and RFP-LC3 puncta was examined by confocal microscopy. Scale bars: 10 μm. GFP or RFP puncta were counted at least in 20 cells. **F** Cells were treated without or with BAF (100 nM) in the presence or absence of 1 μM TSN for 24 h, the expression of SQSTM1 and LC3-II was analyzed by western blot. Comparisons of the intensities were statistically estimated and represented as mean ± SD for three independent experiments (*p < 0.05, **p < 0.01, ***p < 0.001 vs. CTL)

MAP1LC3/LC3 (microtubule-associated protein 1 light chain 3) could transform from the LC3-I into its lipid counterpart LC3-II during autophagosome maturation [17]. LC3-II and the cargo adaptor protein, p62/SQSTM1 (sequestosome 1) are two widely used markers to monitor autophagy. We examined the effect of TSN (Fig. 1A) on LC3-II and SQSTM1 protein levels in both MDA-MB-231 and MDA-MB-436 cells. Western blot analysis showed that TSN resulted in dose- and time-dependent accumulation of LC3-II and SQSTM1 in both cell lines (Fig. 1B, C), suggesting that TSN might inhibit autophagic maturation. Furthermore, we analyzed fluorescent images of transiently GFP-LC3-expressing MDA-MB-231 cells after treatment of TSN via Leica Microscope. As shown in Fig. 1D, treatment of TSN at 1 μM increased GFP-LC3 puncta dramatically in MDA-MB-231 cells. To confirm whether TSN interrupted autophagosome maturation, we applied the tandem RFP-GFP-LC3 reporter to monitor the autophagy flux. RFP-GFP-LC3 is comprised of two tandem fluorescent proteins: a pH-insensitive RFP and a pH-sensitive GFP. GFP fluorescence will be quenched in an acid environment, leaving an RFP signal only. The ratio of red to green fluorescence allows for a distinction between lysosomal and cytoplasmic LC3, thus could reflect the autophagy flux. The result showed that TSN markedly increased the number of yellow puncta in both MDA-MB-231 and MDA-MB-436 cells (Fig. 1E), similar to that induced by CQ, a classic autophagy inhibitor that impairs autophagosome fusion with lysosomes [18]. In contrast, many red puncta were observed in Torin1-treated cells. Bafilomycin A1 (BAF) is an inhibitor of autophagic flux by inhibiting the acidification of lysosomes. Our results showed that co-incubation of cells with TSN and BAF did not induce the further increase in LC3-II levels compared with TSN treatment alone (Fig. 1F), confirming that TSN inhibits the autophagic degradation. Overall, these observations indicated that TSN was a potent late-stage autophagic flux inhibitor.

### TSN did not inhibit the fusion process of autophagosome and lysosome

**Fig. 2:**
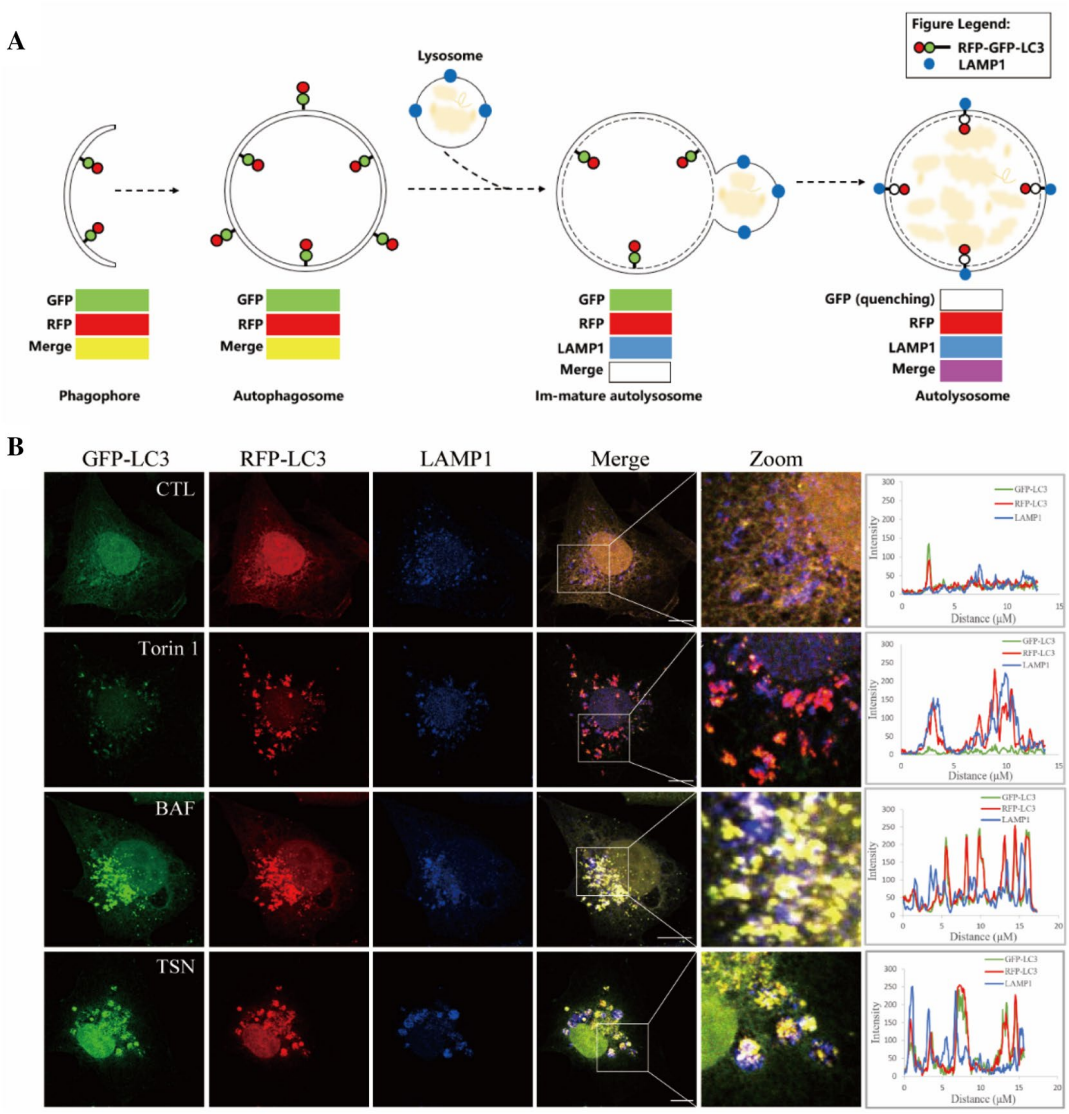
TSN did not inhibit the fusion process of autophagosome and lysosome. **A** Schematic diagram of RFP-GFP-LC3 reporter/LAMP1 reflected the fusion of autophagosome and lysosome. **B** Immunofluorescence photographs of the co-localization of LAMP1 (blue) and stably-expressing RFP-GFP-LC3 MDA-MB-231 cells treated with TSN (1 μM), Torin1 (100 nM), BAF (100 nM) or DMSO for 12 h. Nuclei were stained with Hoechst 33258. Scale bar: 10 μm

It is essential to determine the inhibitory effect of TSN on autophagy is due to the blockade of autophagosome–lysosome fusion, or impairment of autolysosome degradation. To address whether TSN affects the fusion process, we examined the co-localization of RFP-GFP-LC3 and LAMP1, a marker for late endosomal and lysosomal membranes. Figure 2A is the schematic diagram of RFP-GFP-LC3 reporter/LAMP1 to reflect the fusion of autophagosome and lysosome. As shown in Fig. 2B, both TSN and BAF treated cells showed nice colocalization of LC3 and LAMP1, indicating that TSN did not affect the fusion of autophagosome and lysosome.

### TSN elevated lysosome pH and impaired lysosomal proteolytic function

**Fig. 3:**
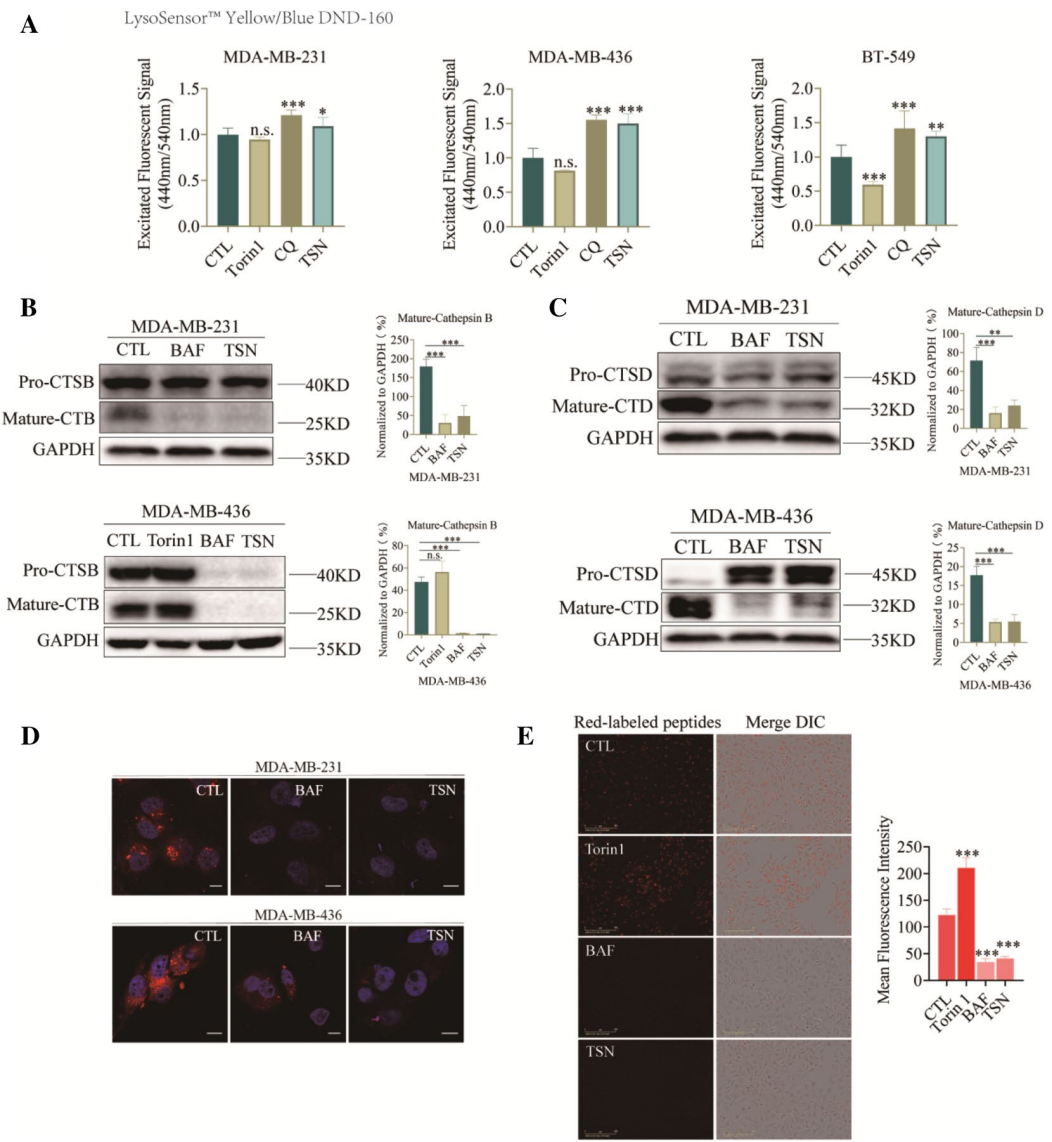
TSN elevated lysosome pH and impaired lysosomal proteolytic function. **A** MDA-MB-231, MDA-MB-436 and BT-549 cells were stained with LysoSensor™ Yellow/Blue DND-160 after treatment of 1 μM TSN, 100 nM Torin1, 30 μM CQ or DMSO for 24 h. The excited fluorescence signal at 440/535 nm was measured by 96-well plate reader. **B**, **C** MDA-MB-231 and MDA-MB-436 cells were treated with 1 μM TSN, 100 nM Torin1, 50 nM BAF or DMSO for 24 h. Total cellular extracts were prepared and subjected to western blot using antibodies against CTSB and CTSD. GAPDH was used as a loading control. **D** Lysotracker with a final concentration of 50 nM was used to label the acid compartment for 30 min. The cells were fixed and stained with Hoechst for further Confocal Scanning. **E** Cells were pre-incubated with 0.01 μg/μL DQ™ Red BSA for 30 min in serum-free DMEM, then treated with 1 μM TSN, 100 nM Torin1, 50 nM BAF or DMSO for 6 h. The images were collected by Incucyte S3 Live-Cell System. The results were the mean ± SD values obtained from three independent experiments (*p < 0.05, **p < 0.01, ***p < 0.001 vs. CTL)

Lysosomes are the acidic organelles (pH 4.5) that digest macromolecules for the regeneration of basic building blocks or defend the invading pathogens. They receive substrates by fusing with endosomes or autophagosomes. To verify whether TSN impacted the function of lysosome thereby blocking the degradation of autophagosome, we used LysoSensor™ Yellow/Blue DND-160, a ratio-metric probe that can be used to measure the pH of lysosomes by calculating the ratio of acquired fluorescent signals emitted at 440/540 nm which were excited at 329/384 nm. As shown in Fig. 3A, the lysosome environment was alkalized after TSN treatment in MDA-MB-231, MDA-MB-436 and BT-549 cells compared with vehicle-treated group. CQ, an autophagy inhibitor by altering the acidic environment of lysosomes, used as a positive control. As the major lysosomal proteases, cathepsins are synthesized as inactive membrane-associated precursors and the precursors are further cleaved to generate active forms within endosomes or lysosomes [19]. We next evaluated whether TSN affected protein expression and the maturation process of cathepsin B (CTSB) and cathepsin D (CTSD). As shown in Fig. 3B, C, TSN dramatically down-regulated the mature form of CTSB and CTSD in MDA-MB-231, and MDA-MB-436 cells, meaning that the cleavage process from inactive to the active form in lysosomes was impeded. A red-fluorescent dye LysoTracker™ Red DND-99 was also used for tracking acidic organelles in live cells. As shown in Fig. 3D, cells almost lose red fluorescence signals after TSN treatment compared with control group. This result further confirmed that TSN increased the pH of lysosome. BAF, a V-ATPase inhibitor well-known to inhibit acidification of lysosome, was used as a positive control. DQ™ Red BSA was also used for proteases function measurement. Upon hydrolysis of the DQ Red BSA to single, dye-labeled peptides by lysosomal proteases, the cell produced brightly fluorescent products. Once the function of the lysosomal proteases is destroyed, a strong fluorescence quenching effect could be observed. As shown in Fig. 3E, TSN treatment led to the red fluorescence quenching, indicating that the lysosomal proteases hydrolytic function was impaired. Taken together, these data indicated that TSN impaired the acid environment of lysosomes and prevented the maturation of lysosomal cathepsin to inhibit the lysosomal hydrolytic function.

### SN-38 induced autophagy in TNBC

**Fig. 4:**
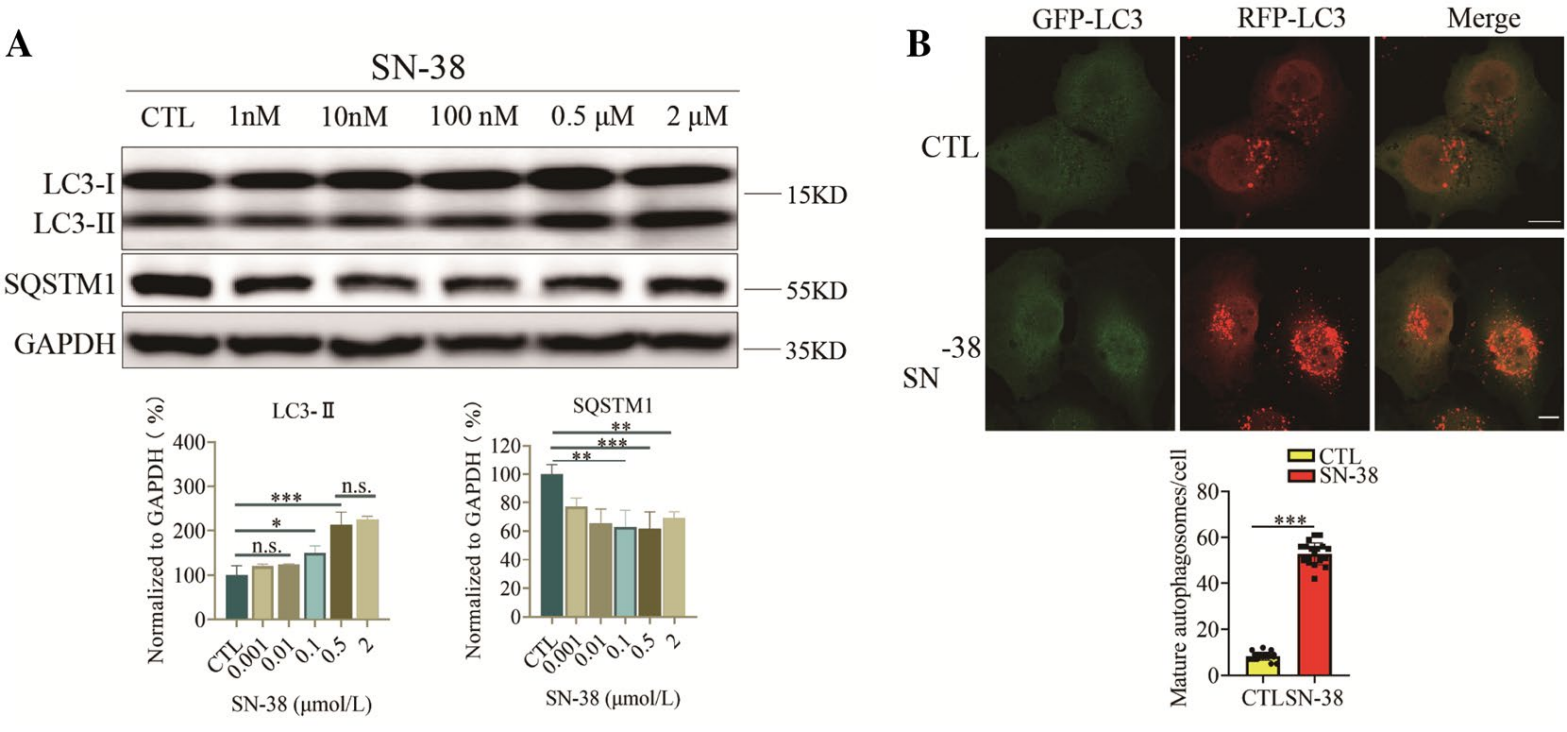
SN-38 induced autophagy in TNBC. **A** Western blot analysis of LC3-II and SQSTM1 levels in MDA-MB-231 cells treated with the indicated concentrations (0.01–1 μM) of SN-38 for 24 h. **B** Cells were transfected with a tandem fluorescent-tagged LC3 (tfLC3) and exposed to SN-38 (0.1 μM) or DMSO treatment. The co-localization of GFP-LC3 and RFP-LC3 puncta was examined by confocal microscope and was counted at least 20 cells. Scale bars: 10 μm. The results are the mean ± SD values obtained from three independent experiments (*p < 0.05, **p < 0.01, ***p < 0.001 vs. CTL)

In response to cytotoxic treatments, tumor cells may activate autophagy to promote survival [20]. Our previous study has shown that chemotherapy compound camptothecin induced protective autophagy in multiple tumors [21]. Irinotecan is a topoisomerase I inhibitor that has been shown to hold great potential in treating TNBC [22]. Irinotecan is a derivative of camptothecin and SN-38 is the active metabolite of irinotecan. We determined change of autophagy flux after SN-38 treatment in TNBC cells. As showed in Fig. 4A, SN-38 dose-dependently induced the increase of LC3-II and decrease of SQSTM1. Then we used RFP-GFP-LC3 MDA-MB-231 cells to monitor autophagy level after SN-38 treatment. We found there was a dramatic increase of red-only puncta after SN-38 treatment, implying that SN-38 activated autophagy process (Fig. 4B). These observations confirmed that TNBC cells up-regulated autophagy levels during chemotherapy.

### Autophagy inhibition in combination with chemotherapy was more effective than monotherapy in reducing viability and inducing apoptosis in TNBC

**Fig. 5:**
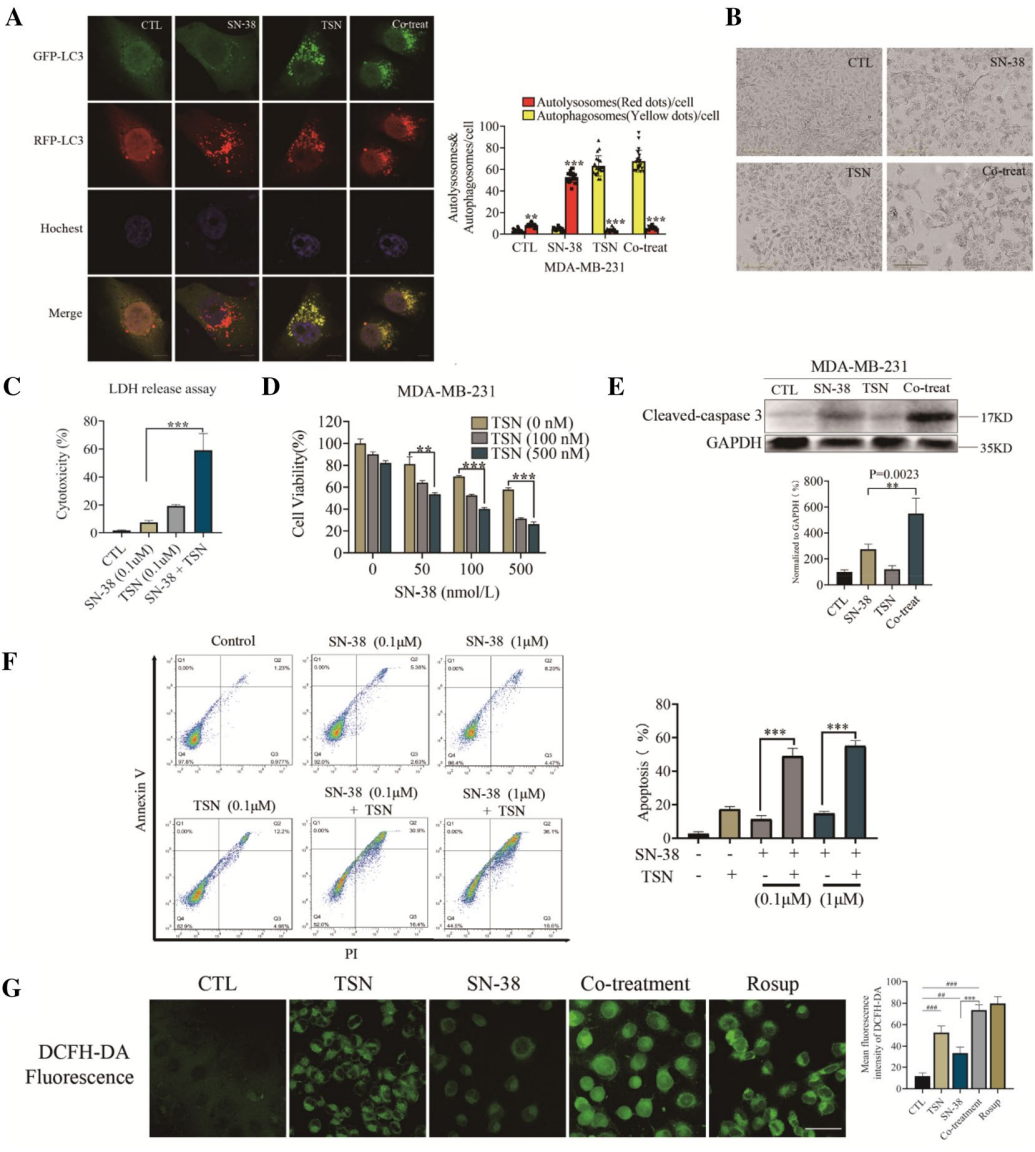
Autophagy inhibition in combination with chemotherapy was more effective than monotherapy in inducing apoptosis in TNBC. **A** Cells were transfected with a tandem fluorescent-tagged LC3 (tfLC3), and were exposed to TSN (1 μM), SN-38 (0.1 μM) or combination. The co-localization of GFP-LC3 and RFP-LC3 puncta was examined by confocal microscopy, in at least 20 cells. Scale bars: 10 μm. **B** MDA-MB-231 cells were treated with 0.1 μM of SN-38 for 24 h, then 0.1 μM TSN was added for another 24 h. Cell morphology was collected by Incucyte S3 Live-Cell System. Scale bars: 200 μm. **C** MDA-MB-231 cells were treated with the indicated concentrations of TSN (0.1 μM) with or without SN-38 (0.1 μM) for 24 h, and the supernatants were collected for the LDH release measurement. **D** MDA-MB-231 cells were treated with various concentrations of SN-38 for 24 h, then TSN was added for another 24 h. MTT assays were performed to assess cell viability. **E** MDA-MB-231 cells were treated with SN-38 (0.1 μM) for 24 h. Then, TSN (0.1 μM) was added into the plate in the presence of SN-38 for another 24 h. PI positive late apoptotic/necrotic cells were used for quantitative analysis. **F** Western blot analysis of cleaved caspase 3 in MDA-MB-231 cells treated with or without SN-38 (0.1 μM), TSN (0.1 μM) for 24 h. **G** MDA-MB-231 cells were treated with or without SN-38 (0.1 μM), TSN (0.1 μM) for 24 h. Then the cells were incubated with DCFH-DA for 20 min at 37 °C, and the fluorescence was observed by confocal laser-scanning microscope (488 nm excitation and 525 nm emission). The results are expressed as the mean ± SD values obtained from three independent experiments (**p < 0.01, ***p < 0.001 vs. SN-38 alone; ^##^p < 0.01, ^###^p < 0.001 vs. CTL)

Increasing evidence indicates that inhibiting autophagy enhances the efficacy of chemotherapy by abolishing chemo-resistance and increasing cancer cell death [23]. We, therefore, determined whether TSN would sensitize breast cancer cells to chemotherapy by blocking protective autophagy. Firstly, we examined the autophagy flux by the RFP-GFP-LC3 reporter. Co-treatment of SN-38 and TSN totally inhibited the maturation of autolysosome showing with yellow puncta after merge (Fig. 5A). Next, we examined whether TSN enhanced the anti-cancer activity of SN-38. Changes in cell morphology showed in Fig. 5B, and autophagy inhibition by TSN rendered TNBC cells more susceptible to chemotherapy. LDH release assay and MTT assay showed that TSN dramatically enhanced the SN-38-induced cell death and growth inhibition (Fig. 5C, D). Next, we performed an Annexin V-FITC/PI double-staining assay using flow cytometric analysis (Fig. 5F). Compared with the SN-38 treated alone cells, co-treatment cells for 48 h increased the proportion of FITC+/PI+ (apoptosis) and FITC−/PI+ (necrosis) cells rate from 11.33% ± 2.08% to 49.10% ± 4.58% (0.1 μM) and 14.86% ± 1.32% to 55.16% ± 3.01% (1 μM), respectively (Fig. 5F). Consistent with these findings, combined treatment of TSN and SN-38 resulted in the increased level of cleaved caspase 3 as shown by western blot (Fig. 5E). Moreover, we examined reactive oxygen species (ROS) release and autophagy inhibition by TSN caused apparently ROS release in co-treatment group. These findings indicated that TSN was able to sensitize TNBC cells to SN-38-induced cell death probably through the suppression of autophagy flux.

### TSN inhibited autophagic flux and repressed TNBC xenograft growth in vivo

**Fig. 6:**
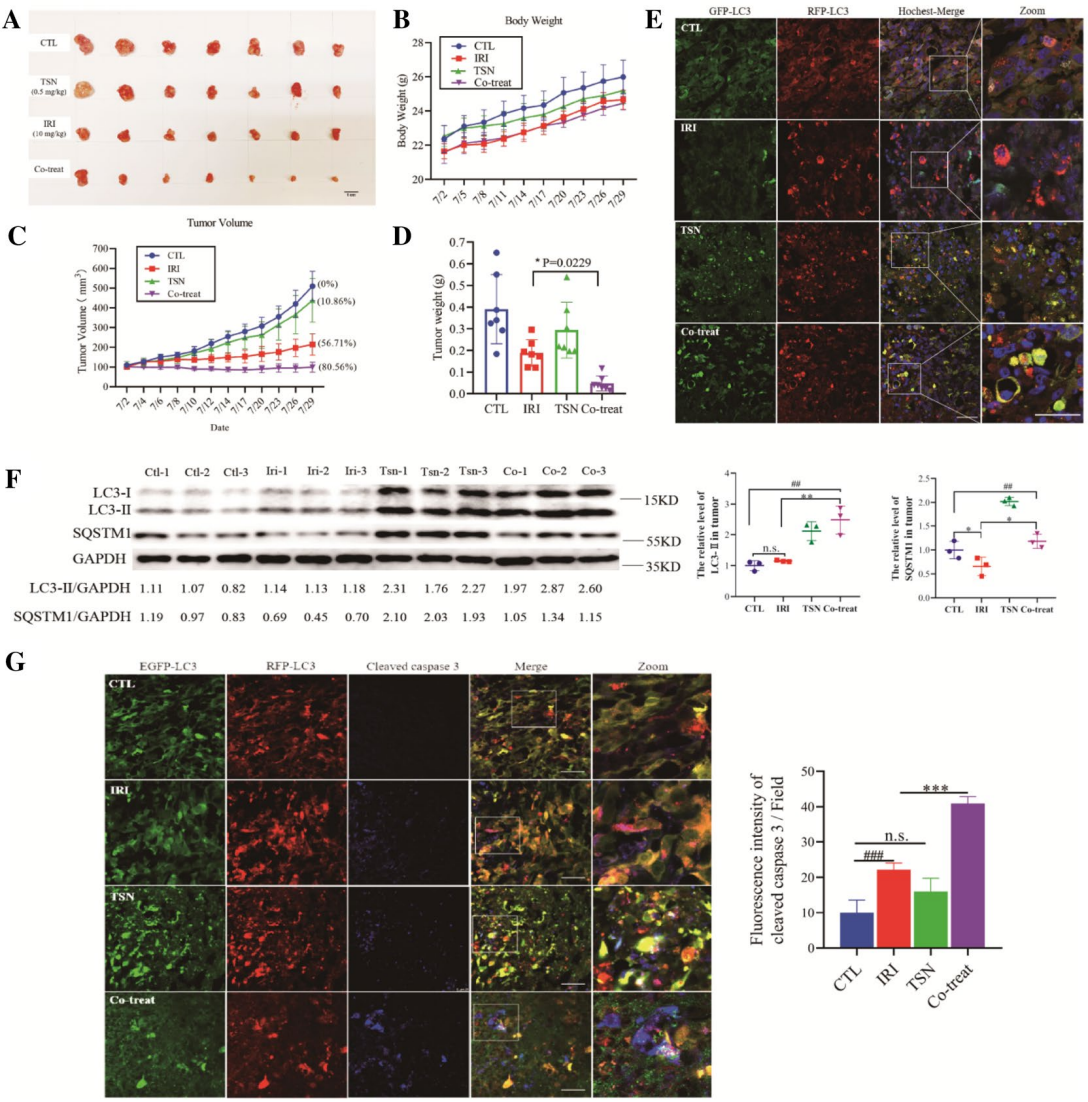
TSN inhibited autophagic flux and repressed TNBC xenograft growth in vivo. **A** Images showed the tumors tissues isolated from mice receiving vehicle, irinotecan (10 mg/kg), TSN (0.5 mg/kg) or SN-38 a combination of irinotecan and TSN treatment. **B** Body weight changes of mice during 28 days of exposure. **C** Average tumor volume calculation. Error bars represent means ± SD. **D** Tumor weight measurement at day 28. **E** Tumor tissues were sectioned and stained with hoechst. The fluorescent images were taken under a confocal microscope. **F** Western blot analysis of LC3-II and SQSTM1 in tumor tissues (n = 3) and the densitometric analysis of LC3-II and SQSTM1 in tumor tissues. **G** Tumor tissues were sectioned and subjected to immunostaining of cleaved caspase 3 for evaluating apoptosis. Scale bars: 100 μm. The results are expressed as the mean ± SD values obtained from three independent experiments (*p < 0.05, **p < 0.01, ***p < 0.001 vs. irinotecan alone; ^##^p < 0.01, ^###^p < 0.001 vs. CTL)

To determine whether our in vitro findings that suppression of autophagy by TSN sensitized TNBC to SN-38-induced cell death could be reproduced in vivo, we examined the therapeutic efficacy of TSN on mouse TNBC xenograft model. To observe the autophagy flux in vivo, nude mice were inoculated subcutaneously with MDA-MB-231 cells stably expressing RFP-GFP-LC3, followed by intraperitoneal administration of irinotecan (10 mg/kg) or TSN (0.5 mg/kg) alone or in combination for 4 weeks. As shown in Fig. 6C, irinotecan treatment alone group showed the inhibitory effect on the growth of tumors with a 56.71% inhibition rate, whereas a combination of TSN and irinotecan led to a significant reduction in tumor growth compared to untreated controls with an 80.56% inhibition rate. Consistently, the tumor weight in the co-treatment group led to a significant reduction (p = 0.0229 for combination treatment vs irinotecan alone) as showed in Fig. 6D. Moreover, no significant changes in body weight were observed among vehicle control, irinotecan or TSN, and a combination of irinotecan and TSN groups (Fig. 6B). The appearance of tumors was consistent with the data of tumor volume and weight (Fig. 6A).

To evaluate whether the combination of TSN and irinotecan results in a change of autophagy flux in xenograft tumor tissue in vivo, tumor samples were excised and stained with hochest. As showed in Fig. 6E, in the basal condition, mature autophagosomes (red-only puncta) were observed in most tumor cells, indicating that induction of autophagy flux under the hypoxia and energy-deficient environment in the TNBC mice. Moreover, in chemotherapy condition (irinotecan group), the number of mature autophagosomes was dramatically increased, meaning that protective autophagy was triggered in tumor tissue. Consistent with the in vitro experiments, TSN treatment showed the blockage of the autophagosome maturation process as evidenced by the markedly increased number of yellow puncta, either in TSN alone or TSN combination with irinotecan group (Fig. 6E). Western blot analysis showed that irinotecan treatment increased the accumulation of LC3-II and decreased the level of SQSTM1 (Fig. 6F). While co-treatment with irinotecan and TSN increased both the levels of LC3-II and SQSTM1 compared with the irinotecan alone group, indicating irinotecan-induced protective autophagy was blocked by TSN. Excised tumor samples were also subjected to cleaved caspase 3 immunostaining. Either TSN or irinotecan treatment alone caused a minimal increase in the number of cleaved caspase 3-positive cells (Fig. 6G) compared with control. However, the irinotecan and TSN combination dramatically increased the number of cleaved caspase 3-positive cells compared with either irinotecan or TSN alone group (Fig. 6G), indicating that autophagy inhibition by TSN rendered cancer cells more vulnerable to chemotherapy. The liver index showing the ratio of organ weight to body weight was analyzed in Table 1. Compared with the control group, IRI, TSN and co-treatment did not appear hepatomegaly and had no significant difference in the liver index of mice (p > 0.05). Together with body weight data, the result was conceivable that at the dosage capable of inhibiting autophagy, the TSN administration was tolerant in mice. Taken together, these findings suggested that TSN potently enhanced the therapeutic activity of irinotecan in vivo by inhibiting autophagy flux.

**Table 1:** Liver mass and liver index^a^ in mice

Group	(Body mass)/g	(Liver mass)/g	(Liver index)/%
CTL	26.26 ± 2.42	1.42 ± 0.24	5.38 ± 0.57
IRI	24.66 ± 1.36	1.26 ± 0.13	5.10 ± 0.50^n.s^
TSN	25.20 ± 1.86	1.43 ± 0.17	5.65 ± 0.47^n.s^
Co-treat	24.36 ± 1.00	1.32 ± 0.10	5.47 ± 0.61^n.s^

^a^Liver index means the ratio of organ wet weight to body mass

^n.s.^No significant difference among administration group and control group

## Discussion

Autophagy has been recognized as an effective mechanism for cancer survival and drug resistance development in multiple cancer types. Early clinical trials have demonstrated the feasibility and potential benefit of inhibiting autophagy in multiple cancer types, including pancreatic cancer, lung cancer, melanoma, and multiple myeloma [24]. Studies have demonstrated that autophagy can be inhibited pharmacologically by targeting the different stages of the autophagic process. Early-stage inhibitors like 3-MA and LY294002, function on class III phosphatidylinositol 3-kinase (PIK3C3) and block the formation of autophagosomes [25, 26]. The inhibitors that target the late stage of autophagy like chloroquine, BAF, leupeptin, etc. impair the fusion of the autophagosome with lysosome or hydrolysis function of lysosome. CQ and HCQ are currently the only clinically available drugs to inhibit autophagy. However, the high effective concentration of CQ and HCQ suggests that they are not highly potent and selective. Therefore, it is necessary to discover more potent and specific autophagy regulators to modulate autophagy in cancer cells.

TNBC remains the most challenging breast cancer subtype to treat. Recently, therapies directed to specific molecular targets have rarely achieved effective improvement during clinical remedy, and chemotherapy remains the standard of treatment. Nonetheless, TNBC patients frequently develop resistance to chemotherapy and it lead to the failure of treatment [4]. Drug efflux (overexpreesion of ATP binding cassette transporters proteins, and P-glycoprotein) [27], up- or down-regulated autophagy flux, de-regulation of distinct cell intrinsic processes (the nuclear proto-oncogene c-MYC) [28], growth factor signaling, and DNA repair have previously been explored as the mechanisms for TNBC chemo-resistance. Therefore, there is still an urgent need to develop novel treatment to improve the therapeutic benefit of chemotherapy, especially for the patients with advanced, chemotherapy resistant TNBC.

TSN, a triterpenoid extracted from *Melia toosendan* Sieb. *et* Zucc, has been reported to possess antioxidant, anti-inflammatory, and anti-allergic activities [29, 30]. Recent studies have revealed potential anti-cancer activity of TSN in diverse cancer models, such as glioblastoma, Ewing’s sarcoma, gastric cancer and hepatocellular carcinoma [29, 31–34]. Tada et al. [35] found that TSN exhibits cytotoxicity against human cancer cells and the toxicity mechanism related to the C-14/C-15 epoxy structure of TSN. Luo et al. [29] proved that TSN significantly inhibited epithelial–mesenchymal transition and migration, and invasion of lung cancer cells. Moreover, He et al. [34] demonstrated that TSN possessed strong anticancer effects in vivo and in vitro via inducing mitochondria-dependent apoptosis in hepatocellular carcinoma cells. These studies provided a possible mechanistic explanation for the anti-tumor effect of TSN. However, the mechanism of action in regards to autophagy flux and lysosome function has not been elucidated. To date, only two study described the relationship between TSN and autophagy in mammal cells [13, 36]. They claimed that TSN developed an apoptosis-sensitizing effect by inducing autophagy in lung cancer cells and TNBC. However, when checking the experiment data, no autophagy flux assay was applied in the study thus the interpretation of the results could be problematic. In our study, we provided a careful characterization of the effect of TSN on autophagy according to Autophagy Modulator Scoring System (AMSS) [37] and autophagy research guideline [38], and confirmed that TSN is a late-stage autophagy inhibitor. Considering the high potency of TSN in inhibiting autophagy both in vitro and in vivo, we believe that autophagy inhibiting activity is an important anti-cancer mechanism of TSN.

TSN has been used in clinics for treating intestinal ascariasis in China [39]. However, liver toxicity of TSN in mice has been reported [40], raising the concern for the use of TSN as an anti-cancer drug. According to the mice data, TSN did not induce obvious liver toxicity at the dosage up to 40 mg/kg (intragastric administration), implying there is still a safety window for the clinic use of TSN. Due to different drug administration routes, we cannot directly compare the dosage we used in the current study (0.5 mg/kg, intraperitoneal administration) with that used in the previous study (40 mg/kg, intragastric administration). However, our data showed that at the dosage sufficient to inhibit autophagy in tumor tissue, TSN did not cause obvious liver toxicity according to the liver index. Nevertheless, extensive safety characterization is still need to evaluate the safety of TSN for long-term application.

Up-regulation of autophagy observed in tumor cells following anti-cancer treatment is regarded as a protective response [41–43], and therapeutic targeting at autophagy might represent a novel molecular avenue to reduce the emergence of chemo-resistance [8]. Irinotecan was initially approved for the first-line treatment of metastatic colorectal cancer and later was also approved for lung cancer treatment [14]. Irinotecan can be suitable for TNBC treatment given the fact that a considerable portion of TNBC tumors harbor mutations in genes required for DNA repairment [22]. Indeed, recent studies highlighted the potential of irinotecan in the BRACness TNBC treatment [22, 44, 45]. In this report, we showed that irinotecan/SN-38 induced autophagy in TNBC cells as a survival mechanism, and autophagy inhibition by TSN sensitized TNBC cells to irinotecan/SN-38 chemotherapy. Taken together, the data generated from this study reveals a novel therapeutic strategy for TNBC treatment by combination of topoisomerase I inhibitor and autophagy inhibitor.

## Conclusion

In summary, our results demonstrate that TSN is a potent late-stage autophagy inhibitor by impairing the lysosome acidification and hydrolases activity. Through autophagy inhibition, TSN could blocked irinotecan/SN-38-induced protective autophagy and enhance the sensitivity of TNBC to irinotecan/SN-38 treatment in vitro and in vivo.

## Supplementary Information

**Additional file 1: Fig. S1.** Examination of key autophagy factors, ATG5 ATG7, and Beclin 1.

## Data Availability

The data analyzed during this study can be obtained from the corresponding author on reasonable request.

## Abbreviations

AMSS: Autophagy Modulator Scoring System
BAF: Bafilomycin A1
BSA: Bovine Serum Albumin Standard
CQ: Chloroquine
CTSB: Cathepsin B
CTSD: Cathepsin D
DMEM: Dulbecco’s modified Eagle’s medium
DMSO: Dimethyl sulfoxide
ER: Estrogen receptor
ERK: Extracellular signal-regulated kinase
GFP: Green fluorescent protein
HCQ: Hydroxychloroquine
HER2: Human epidermal growth factor receptor 2
IRI: Irrinotecan
LAMP1: Lysosomal associated membrane protein 1
LC3: Microtubule associated protein 1 light chain 3
LDH: Lactate dehydrogenase
MAPK14: Mitogen-activated protein kinase 14
MTT: 3-(4,5-Dimethylthiazol-2-yl)-2,5-diphenyl-2H-tetrazolium bromide
OCT: Optimal cutting temperature
O.D.: Optical density
PI: Propidium iodide
PIK3C3: Class III phosphatidylinositol 3-kinase
PR: Progesterone receptor
PVDF: Polyvinylidene difluoride
RFP: Red fluorescent protein
RIPA: Radio-immunoprecipitation assay buffer
RT: Room temperature
SN-38: 7-Ethyl-10-hydroxycamptothecin
SQSTM1: Sequestosome 1
TBST: Tris-buffered saline with 0.1% Tween-20
tfLC3: Tandem fluorescent-tagged LC3
TNBC: Triple-negative breast cancer
TSN: Toosendanin
TUNEL: TdT-mediated dUTP nick-end labeling
