# Supplementary material for: Toosendanin, a late-stage autophagy inhibitor, sensitizes triple-negative breast cancer to irinotecan chemotherapy
Source: Chin Med. 2022 May 6;17:55. doi: 10.1186/s13020-022-00605-8 (PMC9074333; doi:10.1186/s13020-022-00605-8)
Supplement: Supplementary file 1 — Additional file 1: Fig. S1. Examination of key autophagy factors, ATG5 ATG7, and Beclin 1. [file 13020_2022_605_MOESM1_ESM.docx]

Additional figures


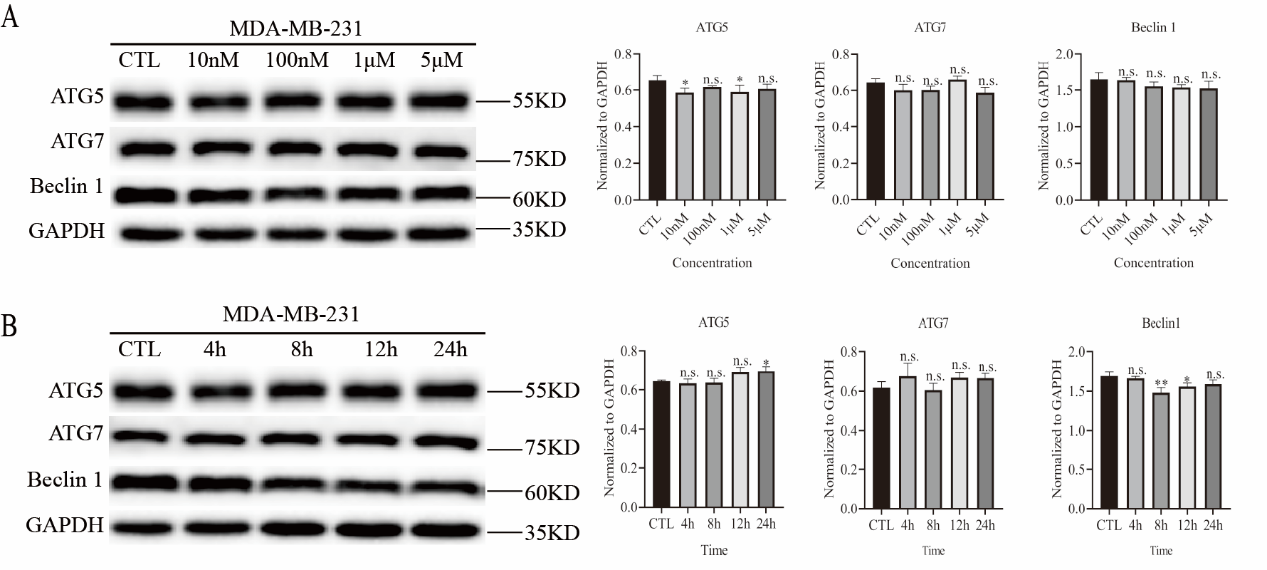


Figure S1. Examination of key autophagy factors, ATG5 ATG7, and Beclin 1. (A) Western blot analysis of ATG5 ATG7, and Beclin 1 levels in MDA-MB-231 cells treated with the indicated concentrations (0.01–5 μM) of TSN for 24 h. (B) Western blot analysis of ATG5 ATG7, and Beclin 1 levels in MDA-MB-231 cells treated with TSN (1 μM) at the time points indicated (0, 4, 8, 12, 24h). Comparisons of the intensities were statistically estimated and represented as mean ± SD for 3 independent experiments (n.s., no significant difference, *p < 0.05, **p < 0.01, ***p < 0.001 vs. CTL).
